# Small Cerebellopontine Angle Meningioma—Surgical Experience of 162 Patients and Literature Review

**DOI:** 10.3389/fonc.2020.558548

**Published:** 2020-10-09

**Authors:** Jiyuan Bu, Pengjie Pan, Hui Yao, Weiyi Gong, Yuan Liu, Zhengquan Yu, Zhong Wang, Jiang Wu, Gang Chen

**Affiliations:** ^1^Department of Neurosurgery & Brain and Nerve Research Laboratory, The First Affiliated Hospital of Soochow University, Suzhou, China; ^2^Department of Neurosurgery, First People’s Hospital of Kunshan, Suzhou, China

**Keywords:** small cerebellopontine angle meningioma, stereotactic radiotherapy, surgical treatment, symptom deterioration, tumor enlargement, further treatments

## Abstract

**Objective:**

To retrospective analyze the clinical data of 162 patients with small cerebellopontine angle meningiomas. To compare with the nature of tumors, symptoms pre- and post-treatments, neurological deficit, and prognosis in literatures. To explore the surgical outcomes of small cerebellopontine angle meningiomas and summarize the surgical experience.

**Methods:**

All of 162 patients with small cerebellopontine angle meningiomas underwent surgery between January 2010 and December 2019 in the neurosurgery department of the First Affiliated Hospital of Soochow University. This cohort of eight literatures reported about stereotactic radiotherapy of small cerebellopontine angle meningiomas between January 2010 and December 2019. All clinical data were obtained for analysis.

**Results:**

Compared with stereotactic radiotherapy, surgical treatment for small cerebellopontine angle meningiomas lead to the better results in relieving symptoms and inhibiting tumor progression. Surgical treatment can obtain the exact pathological examination results to guide the further treatment.

**Conclusions:**

Surgical treatment should be the first choice for small cerebellopontine angle meningiomas.

## Introduction

Cerebellopontine angle meningiomas account for 6–15% of the tumors in the cerebellopontine angle region (1). They are characterized by the deep tumor location, narrow surgical field, and proximity to the brainstem, multiple pairs of (V–XI) cranial nerves (2). At present, surgical treatment is the first choice for large cerebellopontine angle meningiomas, while small cerebellopontine angle meningiomas are always treated by stereotactic radiotherapy, pharmacotherapy and experimental therapy (3). In recent years, many studies have found that stereotactic radiotherapy had the limitations of low tumor control rate, post-treatment brain edema, and tissue adhesion, which hindered the further treatments (4). Therefore, more and more small cerebellopontine angle meningioma patients are turning to surgical treatment.

## Materials and Methods

### Study Design

A retrospective analysis of small cerebellopontine angle meningioma patients was performed. These patients were operated on in the neurosurgery department of the First Affiliated Hospital of Soochow University between January 2010 and December 2019. These patients were classified as the surgery group. We also reviewed the literature on stereotactic radiotherapy of small cerebellopontine angle meningiomas from the past 10 years. The clinical data of patients in the literature were collected and analyzed, and these patients were classified as the radiotherapy group. We analyzed the differences between the two groups, including the nature of tumors, symptoms pre- and post-treatments, neurological deficits, and prognosis. Finally, we explored the surgical outcomes of small cerebellopontine angle meningiomas and summarized the surgical experience.

### Inclusion Criteria

#### Diagnosis

To distinguish meningiomas from other cerebellopontine angle tumor, such as acoustic neuromas and gliomas, all the patients were diagnosed by both radiological and histopathological examination, including MRI, CT, PET, and SSTR2 ligands. Both the dural tail sign on MRI and no expansion of internal auditory canal on CT are the main differential points between cerebellopontine angle meningiomas and acoustic neuromas. To differentiate cerebellopontine angle meningiomas from gliomas and metastases, patients were conventionally tested by MR spectroscopy (5). In terms of histological aspects, immunohistochemical analysis was also conventionally tested, such as HE staining, Vimentin staining, EMA staining, Ki-67 and CD56.

#### Surgery Group

(1) The patients with small cerebellopontine angle meningioma were operated between January 2010 and December 2019, (2) tumor volume ≤ 8 cm^3^, (3) no related treatment before surgery, (4) no other nervous system diseases, and (5) kept in touch during follow-up.

#### Radiotherapy Group

(1) The patients with small cerebellopontine angle meningioma were collected from the literature about stereotactic radiotherapy, which published between January 2010 and December 2019, (2) the included patients had complete pre- and post-treatment data, (3) no other nervous system diseases, and (4) kept in touch during follow-up.

Follow-up consisted of routinely visiting the patients and performing MRI or CT tests every 3–6 months for the first 3 years after treatment, and then visiting and testing every year.

### Classification of Tumors

**FIGURE 1:**
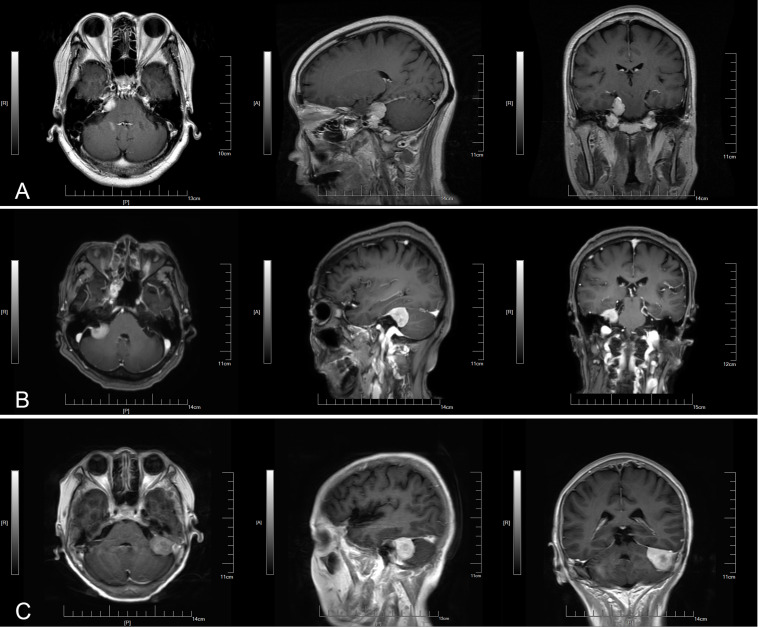
Three types of small cerebellopontine angle meningiomas. **(A)** Anterior. **(B)** Middle. **(C)** Posterior.

Based on the central site of dural attachment, cerebellopontine angle meningiomas were classified into three types (6): anterior tumors were those that originated from the tentorium cerebelli or the petrous bone dura anterior the internal auditory canal; middle tumors were those that originated from the dura mater in the internal auditory canal; posterior tumors were those that originated from the sigmoid and transverse sinuses or the petrous bone dura posterior the internal auditory canal (Figure 1).

Based on tumor pathology, cerebellopontine angle meningiomas were classified into three grades: WHO grade I (low recurrence and low invasive growth), WHO grade II (high recurrence and high invasive growth), and WHO grade III (strong recurrence and metastasize systemically).

Based on the grade of tumor resection, patients with cerebellopontine angle meningiomas were divided into five grades: Simpson grade I: total resection of the meningioma, dural attachment, and skull; Simpson grade II: total resection of the meningioma and electrocoagulation or laser treatment with dural attachment; Simpson grade III: total resection of meningiomas and no treatment with the dural attachment and skull; Simpson grade IV: partial resection of meningiomas; Simpson grade V: decompression and tumor biopsy.

Tumor progression and regression were defined as volume changes of more than 15% on radiological examination.

### Surgical Program

All of the patients were using the suboccipital retrosigmoid approach. A suboccipital retrosigmoid straight incision of about 8–10 cm was made on the affected side. The long diameter of the oval bone window was 5 cm, and the short diameter was 3.5 cm. The bone window up to the transverse sinus, lateral to the mastoid root, exposed the angle between the sigmoid and transverse sinus. Cut the dura, stretched the cerebellum, opened up the cisterna magna, and released the cerebrospinal fluid. Finally, the cerebellopontine angle meningioma was completely resected, taking care to protect the petrous vein, the trigeminal nerve, and the abducens nerve. During the operation, electrophysiological monitoring was used to monitor the facial and acoustic nerves.

### Statistical Analysis

All of the statistical analyses were performed with SPSS version 22.0. The parametric continuous variables were reported as mean ± standard deviation. The non-parametric variables were reported as the median with the range. Clinical outcomes and signs and symptoms were reported as three-category data (improvement or enlargement, no change, deterioration or diminution). The independent samples *t*-test was performed for two categories of data, and ANOVA was performed for three-category data. The chi-square test was performed to compare nominally distributed categorical variables. Logistic regression analysis was performed for multivariate analyses. *P*-values less than 0.05 were considered to be statistically significant.

## Results

### Surgery Group

#### Participants

A total of 162 patients with small cerebellopontine angle meningioma were included, including 53 males and 109 females, with an average age of 54.85 years (21–89 years). The preoperative symptoms were headache and dizziness (96), hearing loss and tinnitus (48), facial sensation and paralysis (29), walking instability and ataxia (28), hoarseness, poor cough reflex (16). There were 77 patients with two or more symptoms and 64 patients without symptoms before diagnosis.

#### The Nature of the Tumors

The volume of tumors ranged from 1.042 to 8.161 cm^3^, with an average of 4.710 cm^3^; 72 patients had anterior tumors, 41 patients had middle tumors, and 49 patients had posterior tumors; 148 patients had WHO grade I tumors, and 14 patients had WHO grade II tumors.

#### Surgical Outcomes

There were 152 cases of Simpson grade I and 10 cases of Simpson grade II, 41 cases of post-operative symptom relief, 110 cases of no significant change, 11 cases of aggravation or new symptoms.

### Radiotherapy Group

A total of 1644 patients with small cerebellopontine angle meningioma were included, including 335 males and 1309 females (Table 1).

**TABLE 1:** Characteristics of patients in literatures.

	Sex (M/F)	Age	Tumor volume	Symptomatic outcomes			Radiologic outcomes			Further treatment	
				No change	Improvement	Deterioration	No change	Diminution	Enlargement	Radiotherapy	Surgery
Kim et al. (13)	34/119	56.6 ± 7.4	2.5 (0.22–3.81)	57	92	4	91	53	9	1	1
Ge et al. (14)	23/107	54.5 (25–81)	3.68 (0.23–4.58)	83	40	7	86	37	7	4	3
Jahanbakhshi et al. (15)	18/75	52.2 (25–79)	6.0 (1.5–7.42)	36	46	11	38	52	3	3	6
Patibandla et al. (16)	28/92	61 (12–88)	4.0 (0.4–6.1)	85	28	7	21	86	13	2	2
Faramand et al. (17)	10/31	61 (39–83)	3.1 (0.3–7.1)	18	16	7	20	15	6	9	5
Sheehan et al. (18)	140/535	57.6 (12–89)	6.5 (0.15–8.14)	424	201	50	275	336	64	10	24
Starke et al. (19)	54/201	55 (19–85)	5.0 (0.3–5.5)	208	22	25	95	125	35	16	14
Ding et al. (20)	28/149	59.2 ± 12.18	3.6 (1.9–6.2)	60	101	16	81	82	14	9	7
Summary	335/1309	–	–	971	546	127	707	786	151	54	62

Of the radiotherapy group, 971 patients (59.1%) had no significant change in symptoms after treatment, 546 patients (33.2%) showed an improvement in symptoms, and 127 patients (7.7%) showed a worsening of symptoms or developed new neurological symptoms.

There were 151 patients (9.2%) in the radiotherapy group with tumor recurrence during follow-up.

There were 54 patients (3.3%) received second stereotactic radiotherapy, and 62 patients (3.8%) underwent surgery during follow-up.

### Symptomatic Outcomes

Of the surgery group, 102 patients (63.0%) had no significant change in symptoms after surgery, 49 patients (30.2%) showed an improvement in symptoms, and 11 patients (6.8%) showed a worsening of symptoms or developed new neurological symptoms. There was no significant difference in the symptom deterioration rate between the surgery group and the radiotherapy group, with the exception of Andrew et al.’s study (Table 2). The symptom deterioration rate of Andrew et al. was significantly higher than that of the surgery group.

**TABLE 2:** Symptom deterioration rate.

	Number	Symptom deterioration	λ^2^ Value	*p* Value
Kim et al. (13)	153	4	3.025	0.082
Ge et al. (14)	130	7	0.246	0.620
Jahanbakhshi et al. (15)	93	11	1.902	0.168
Patibandla et al. (16)	120	7	0.106	0.745
Faramand et al. (17)	41	7	4.281	0.039*
Sheehan et al. (18)	675	50	0.074	0.786
Starke et al. (19)	255	25	1.141	0.286
Ding et al. (20	177	16	0.584	0.445
Surgery group	162	11	–	–

*Statistically significant (*p* < 0.05).

### Radiologic Outcomes

There were 10 patients (6.2%) in the surgery group with tumor recurrence during follow-up. We found no significant difference in tumor enlargement rate between the surgery group and the radiotherapy group, with the exception of Robert et al. (Table 3). The tumor enlargement rate of Robert et al. was significantly higher than that of the surgery group.

**TABLE 3:** Tumor enlargement rate.

	Number	Tumor enlargement	λ^2^ Value	*p* Value
Kim et al. (13)	153	9	0.012	0.914
Ge et al. (14)	130	7	0.082	0.775
Jahanbakhshi et al. (15)	93	3	1.061	0.303
Patibandla et al. (16)	120	13	1.999	0.157
Faramand et al. (17)	41	6	3.226	0.073
Sheehan et al. (18)	675	59	1.139	0.286
Starke et al. (19)	255	35	5.870	0.015*
Ding et al. (20)	177	14	0.388	0.533
Surgery group	162	10	–	–

*Statistically significant (*p* < 0.05).

### Further Treatment

In the surgery group, there was one patient (0.6%) who underwent a second resection, and 12 patients (7.4%) received stereotactic radiotherapy during follow-up. We found no significant difference in the further treatment rate between the surgery group and the radiotherapy group, except for Kyung et al. and Andrew et al. (Table 4). The further treatment rate of Kyung et al. was significantly lower than that of the surgery group, while the further treatment rate of Andrew et al. was significantly higher than that of the surgery group.

**TABLE 4:** Further treatment.

	Number	Further treatment	λ^2^ Value	*p* Value
Kim et al. (13)	153	2	7.829	0.005*
Ge et al. (14)	130	7	0.788	0.375
Jahanbakhshi et al. (15)	93	9	0.205	0.651
Patibandla et al. (16)	120	4	2.678	0.102
Faramand et al. (17)	41	14	19.36	<0.0001*
Sheehan et al. (18)	675	34	2.200	0.139
Starke et al. (19)	255	30	1.498	0.221
Ding et al. (20)	177	16	0.111	0.739
Surgery group	162	13	–	–

*Statistically significant (*p* < 0.05).

In the surgery group, WHO grade II and Simpson grade II were risk factors of further treatment (Table 5).

**TABLE 5:** Risk factors of further treatments.

Risk factors	*p* Value	OR	95% CI
WHO II grade	0.045	2.504	0.108–58.24
Simpson II grade	0.001	20.34	0.829–49.90

## Discussion

The choice of surgery or stereotactic radiotherapy depends on the general situation of patients and the nature of tumors (7). Most of cerebellopontine angle meningiomas belong to benign tumors. The surgical effect of cerebellopontine angle meningiomas is always satisfied, and the rates of both post-operative symptom deterioration and tumor enlargement are lower than other nervous system tumors. However, the compression of the brain stem and cerebellum is a frequent occurrence of cerebellopontine angle meningiomas, especially in patients with large cerebellopontine angle meningiomas, the high intracranial pressure can lead to herniation and acute hydrocephalus. Either complete or partial resection can significantly reduce the risk of complications. Hence, surgery is the best choice for patients with large cerebellopontine angle meningiomas. For small cerebellopontine angle meningiomas, stereotactic radiotherapy, including Gamma Knife, cyber knife and other types of linear accelerator (8), is universally acknowledged as the first choice. With the development of medical treatment and the popularization of MRI, the early diagnosis of cerebellopontine angle meningioma in the small-volume or asymptomatic stage turns to possible. Early diagnosis and treatment greatly improve the prognosis of cerebellopontine angle meningioma, as well as bringing a confusion of choice between surgery and stereotactic radiotherapy for small cerebellopontine angle meningioma. In consideration of the edema and adhesion of brain tissue after stereotactic radiotherapy, which hindered the further surgery, more and more studies have supported early surgical treatments.

**FIGURE 2:**
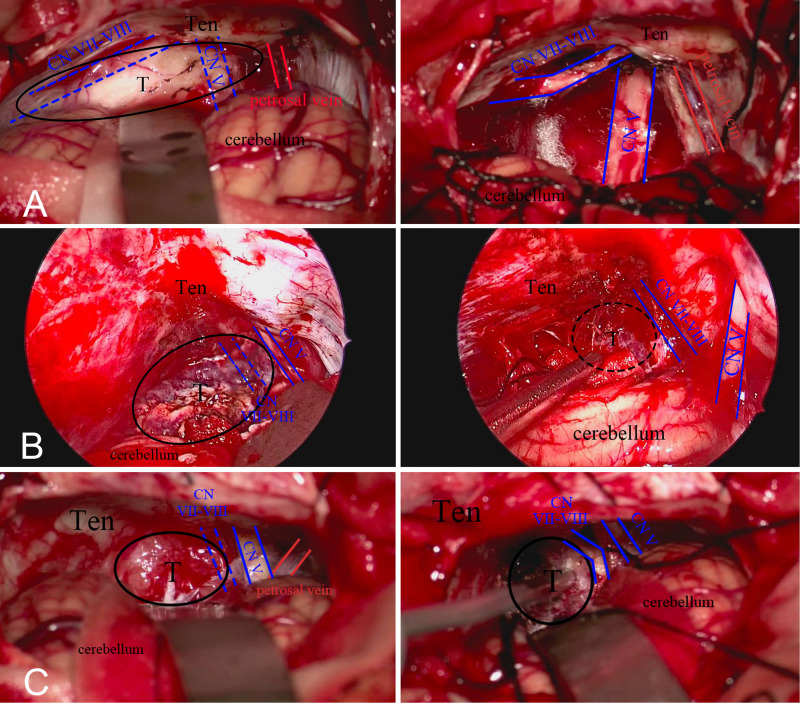
**(A)** Anterior tumors push the facial and auditory nerve to the lateral side. **(B)** Middle tumors push nerve to the ventral aspect. **(C)** Posterior tumors push nerve to the medial inferior side.

In terms of relieving pre-operative symptoms, the symptom deterioration rate in surgery group was similar to or even lower than the rate in radiotherapy group. Compared with stereotactic radiotherapy, surgical treatment can sometimes lead to better results in relieving pre-operative symptoms. Edema of peripheral brain tissue is a common side effect of stereotactic radiotherapy. Swelling of brain tissue will aggravate the tension and compression of nerves, which is the reason why the symptoms become worse after stereotactic radiotherapy. In order to reduce nerve injury and relieve symptom deterioration, operators need to carefully protect brain tissue, nerves, and blood vessels during the surgery. Compared with regular cerebellopontine angle meningiomas, surgical operation on small cerebellopontine angle meningiomas requires more protection for nerves and blood vessels, and neuroelectrophysiological monitoring during the whole surgery process is deemed essential, which contributes to the lower symptom deterioration rate in the surgery group. The most common clinical manifestation of small cerebellopontine angle meningiomas is functional defects of the facial and auditory nerves. Hence, protecting the facial and auditory nerves is a key point of surgery. Different types of cerebellopontine angle meningiomas will push the facial and auditory nerves to different positions (9). Thus, the first step of small cerebellopontine angle meningiomas surgery is locating the facial and auditory nerves. Anterior tumors generally push the facial and auditory nerves to the lateral or lateral inferior side. Posterior tumors generally push the facial and auditory nerves to the medial or medial inferior side. Middle tumors generally push the facial and auditory nerves vertically (Figure 2). Due to the compression of the tumor, the facial and auditory nerves are often elongated and become thin and discolored.

During the surgery, the operator needs to insist on sharp separation when separating the tumor from the facial and auditory nerves. The traction of the facial and auditory nerves and cerebellum should be minimized. The nutrient vessels around the facial and auditory nerves should be preserved as much as possible. Although the facial and auditory nerves are anatomically preserved after surgery, the loss of nerve function still exists in many patients, which may be result from the invasive growth of tumors, intraoperative traction, and heat conduction injury caused by electrocoagulation (10). Effective intraoperative neuroelectrophysiological monitoring can lessen the neuron injury by enabling the timely location of the facial and auditory nerves, thus increasing the rate of nerve function preservation.

In terms of inhibiting tumor progression, the tumor recurrence rate in the surgery group is similar to or even lower than the tumor enlargement rate in the radiotherapy group. Compared to stereotactic radiotherapy, surgical treatment can lead to the better results in inhibiting tumor progression. Stereotactic radiotherapy, mainly referring to Gamma Knife and cyber knife, suppresses tumor progression by killing tumor cells. The target of stereotactic radiotherapy is generally located in the center of tumors, and its dose decreases with the distance away from the center of tumors. Although peripheral dose enhancement technology has emerged in recent years, the problem of incomplete tumor boundary inactivation still exists, which also leads to the increase of the tumor enlargement rate in the radiotherapy group (11). For the surgical treatment of small cerebellopontine angle meningiomas, total resection of the tumor, dural attachment, and skull are key to preventing tumor recurrence. In the surgical principle, the operator needs to resect the dural attachment and skull after the tumor resection to achieve Simpson grade I. When the dural attachment and skull were resected incompletely, electrocoagulation is essential to reduce the possibility of tumor recurrence and achieve Simpson grade II. With the development of radiofrequency laser scalpels and other microinstruments, the surgical treatment of dural attachment and skull resection is becoming more and more standardized. The overall tumor resection rate of small cerebellopontine angle meningiomas is increasing, and the tumor recurrence rate is decreasing year by year.

Many radiotherapy studies have reported that the recurrence of small cerebellopontine angle meningiomas is connected with the pathology of the tumors. WHO grade II meningiomas are more likely to recur than WHO grade I meningiomas, which is related to the characteristic of high invasive growth. However, stereotactic radiotherapy cannot obtain the tumor tissue to examine the pathology of the tumor directly. Therefore, the grade of cerebellopontine angle meningiomas treated with radiotherapy is mostly inferred from imaging examination, which is uncertain. Contrary to stereotactic radiotherapy, surgery can directly obtain tumor tissue for pathological examination and guide further treatment through the exact pathological examination results. In the present study, WHO grade II and Simpson grade II were the risk factors of tumor recurrence after surgery. Therefore, patients with WHO grade II or Simpson grade II who undergo surgery need to receive further treatments at the early stage instead of waiting for the recurrence of tumors. This is also the reason why the recurrence rate in the surgery group was slightly lower than the tumor enlargement rate in the radiotherapy group.

In terms of further treatments, the further treatment rate in the surgery group was similar to or even lower than that reported in the radiotherapy group. Hence, compared with stereotactic radiotherapy, patients with surgical treatment might have a lower likelihood of further treatments. In Kyung et al.’s study, the subjects were asymptomatic patients with small cerebellopontine angle meningiomas. A lack of symptoms or relatively mild symptoms could significantly reduce the subjective desire of patients for treatment, which may be why the further treatment rate in Kyung et al. was lower than that reported in the surgery group. In the radiotherapy group, there was no significant difference between the number of patients who chose further radiotherapy and the number of patients who turned to surgery. In the surgery group, there was 1 patient (0.6%) who underwent a second resection, and 12 patients (7.4%) received stereotactic radiotherapy during follow-up. The reason for further radiotherapy for patients in the surgery group was the high invasiveness of the tumor and the incomplete treatment of the dural attachment and skull (12). Similar to other meningiomas, stereotactic radiotherapy of small cerebellopontine angle meningiomas is more likely to be an auxiliary treatment for inhibiting tumor progression after surgery.

The surgical complications mainly included dysfunction of the facial and auditory nerves, trigeminal nerve, and posterior cranial nerves as well as hydrocephalus. Facial paralysis, facial numbness, and hearing loss can seriously affect the quality of life of patients after surgery. The symptoms of posterior cranial nerve damage, such as hoarseness, dysarthria, and weakened cough reflex, significantly affect the prognosis of patients after surgery. Because of the small size of the tumor, complications, such as hydrocephalus and intracerebral hemorrhage, are rare in patients with small cerebellopontine angle meningiomas.

## Conclusion

Compared with stereotactic radiotherapy, surgical treatment for small cerebellopontine angle meningiomas can sometimes lead to better results in relieving pre-operative symptoms and inhibiting tumor progression. In terms of further treatments, compared with the uncertainty of stereotactic radiotherapy, surgical treatment can obtain exact pathological examination results to guide the further treatment. Similar to large tumors, surgical treatment should be the first choice for small cerebellopontine angle meningiomas, while stereotactic radiotherapy, pharmacotherapy and experimental therapy are more suitable as supplement to surgical treatment.

## Data Availability Statement

The raw data supporting the conclusions of this article will be made available by the authors, without undue reservation.

## Ethics Statement

The studies involving human participants were reviewed and approved by the Institutional Review Board and Ethics Committee of the First Affiliated Hospital of Soochow University. The patients/participants provided their written informed consent to participate in this study.

## Author Contributions

JW and GC designed the study. JB, PP, HY, WG, YL, ZY, and ZW performed the experiments and analyzed the data. JB wrote the manuscript. All the authors read and approved the manuscript. JB, PJ, and HY have contributed equally to this work. All authors contributed to the article and approved the submitted version.

## Conflict of Interest

The authors declare that the research was conducted in the absence of any commercial or financial relationships that could be construed as a potential conflict of interest.
